# Preprocessing of Iris Images for BSIF-Based Biometric Systems: Binary Detected Edges and Iris Unwrapping

**DOI:** 10.3390/s24154805

**Published:** 2024-07-24

**Authors:** Arthur Rubio, Baptiste Magnier

**Affiliations:** 1 Department of Computer Science & Artificial Intelligence, IMT Mines Ales, Ales, France; 2 EuroMov Digital Health in Motion, Univ Montpellier, IMT Mines Ales, Ales, France; 3 Service de Médecine Nucléaire, Centre Hospitalier Universitaire de Nîmes, Université de Montpellier, Nîmes, France

**Keywords:** iris biometric recognition, image preprocessing, Canny algorithm, iris unwrapping, binarized statistical image features, Hough transform, computer vision

## Abstract

This work presents a novel approach to enhancing iris recognition systems through a two-module approach focusing on low-level image preprocessing techniques and advanced feature extraction. The primary contributions of this paper include: (i) the development of a robust preprocessing module utilizing the Canny algorithm for edge detection and the circle-based Hough transform for precise iris extraction, and (ii) the implementation of Binary Statistical Image Features (BSIF) with domain-specific filters trained on iris-specific data for improved biometric identification. By combining these advanced image preprocessing techniques, the proposed method addresses key challenges in iris recognition, such as occlusions, varying pigmentation, and textural diversity. Experimental results on the Human-inspired Domain-specific Binarized Image Features (HDBIF) Dataset, consisting of 1892 iris images, confirm the significant enhancements achieved. Moreover, this paper offers a comprehensive and reproducible research framework by providing source codes and access to the testing database through the Notre Dame University dataset website, thereby facilitating further application and study. Future research will focus on exploring adaptive algorithms and integrating machine learning techniques to improve performance across diverse and unpredictable real-world scenarios.

## 1. Introduction

The field of biometric recognition has rapidly evolved, becoming a cornerstone in both security and personal identification domains. Among various biometric modalities, iris recognition stands out due to its high reliability and uniqueness [1]. Similarly to fingerprints, each individual’s iris pattern offers a complex and distinctive structure, making it an ideal biometric marker. The iconic case of Sharbat Gula [2]—the famous photo of the “Afghan Girl”, whose iris image became emblematic in biometric identification [3], underscores the significance of iris recognition in the modern world. The motivation behind this study lies in harnessing these unique attributes using advanced image preprocessing techniques to enhance the accuracy and efficiency of iris recognition systems.

Recent advancements in biometric systems have integrated these technologies into security infrastructures more prevalently [4], necessitating robust and accurate recognition algorithms. Iris recognition has seen widespread adoption due to its non-intrusiveness and high resistance to deception [5]. However, the preprocessing and recognition of iris images pose significant challenges, primarily due to variations in image quality, lighting conditions, and the position of the eye in space.

Additionally, the application of iris recognition has expanded beyond identification to include other attributes such as gender classification [6], demonstrating the potential of using the same iris code for both recognition and gender classification.

This study introduces an innovative approach that combines two powerful techniques: the Canny edge detection algorithm [7] for precise iris segmentation and Binary Statistical Image Features (BSIF) [8] for effective iris pattern recognition. The strengths of the proposed method lie in its robustness and adaptability. The novel features of this method, such as the use of domain-specific BSIF filters trained on iris-specific data and the integration of a tolerance parameter in the Hough transform, offer significant advantages over existing methods. These enhancements address key challenges in iris recognition, including occlusions, varying pigmentation, and textural diversity, leading to improved accuracy and speed. An applicable scenario for this work involves combining it in parallel with deep learning algorithms [9] to enhance their robustness.

Furthermore, the development of this methodology also serves an educational purpose, providing insights into practical applications of image processing techniques and algorithms in biometric recognition.

## 2. Source Code

The reviewed source code and documentation associated with the proposed algorithms are available from the web page of this article, as well as on the Iris-Images-Preprocessing-for-BSIF-Iris-Recognition (https://github.com/arthur-ru/Iris-Images-Preprocessing-for-BSIF-Iris-Recognition, accessed on 16 July 2024) public GitHub repository. The correspondences between algorithms and source codes are mutually given and the compilation with usage instructions is included in the README.md of the repository. The repository also contains the scripts allowing for the reproduction of the experiments included in various figures.

## 3. Methodology

### 3.1. Overview

**Figure 1:**
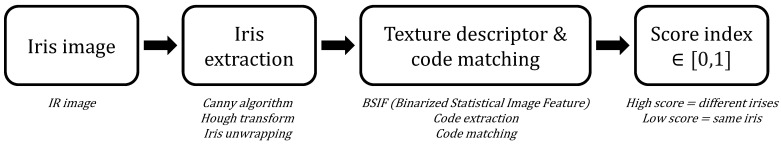
Steps of the process for iris extraction and comparison.

The proposed methodology is divided into two principal modules, each playing a crucial role in the overall process of iris biometric recognition. The different steps are summarized in Figure 1. These modules are designed to efficiently process iris images, extracting relevant information and comparing them for accurate identification. The 1892 images originate from the WACV-2019 Human-inspired Domain-specific Binarized Image Features (HDBIF) Dataset [10]. More details about the dataset are reported in  Table 1.

### 3.2. Module 1: Preprocessing of Iris Images

The first module focuses on the preprocessing of iris images. This process is essential for enhancing the quality and consistency of the images prior to recognition, significantly increasing the accuracy of identification. The preprocessing includes several key steps:

(1) **Image Cleaning**: Raw iris images are first cleaned to remove the noise and undesirable artifacts and enhance clarity. This step is crucial for ensuring the accuracy of subsequent stages and is conducted with a Gaussian and median filter applied to the image, followed by histogram adjustment.
(2) **Edge Detection with the Canny Algorithm**: the Canny algorithm [7], a widely-used method for edge detection, is employed to accurately identify the edges of the iris.
(3) **Hough transform**: We use the Hough transform [11] to find circles for both the pupil and the iris. We start with the pupil since it is usually clear of eyelash interference. After locating the pupil, we cop the image and introduce a tolerance parameter representing the maximum permissible distance between the centers of the pupil and iris (given their close alignment). Then, we use another Hough transform to detect the iris/sclera boundary. Finally, we extract the iris by applying a binarized filter that captures the area between the two detected circles.
(4) **Iris Unwrapping**: This step transforms the iris from its natural annular shape into a rectangular form. This transformation facilitates further analyses and lets the processed image be used for iris recognition using BSIF.
(5) **Mask creation**: The unwrapped iris is thresholded to create a custom mask, which will allow the iris comparison module to not compare the part of the iris covered by eyelids or eyelashes.

### 3.3. Module 2: Iris Recognition

The second module involves iris recognition. After preprocessing, the images are ready for detailed analysis and comparison. This module is based on the work of 4 searchers [12]:

(1) **Feature Extraction with BSIF**: Binary Statistical Image Features (BSIF) are used for extracting the unique characteristics of each iris. These characteristics serve as a biometric signature. In this case, instead of extracting random textures, we will use textures that have been specifically chosen by a team who manually compared irises to determine if they belonged to the same person.
(2) **Comparison and Matching**: Extracted iris codes are compared to determine if they match known samples. This step employs advanced techniques to measure the similarity between images, accounting for possible variations such as eye rotation. Given the number returned (between 0 and 1), you can assess if the two eyes belong to the same person or not.

Together, these two modules form a robust system for iris biometric recognition, offering high precision and reliability in personal identification and security applications. To process and compare irises in our code, we used the HDBIF Dataset. Please follow the instructions here (https://cvrl.nd.edu/projects/data/#wacv-2019-hdbif-dataset, accessed on 16 July 2024) to get a copy of the test database.

## 4. Preprocessing of Iris Images

The preprocessing of iris images is a crucial step in the proposed methodology; without it, the method may diverge or yield inconsistent results. It corresponds to the part where we take an image from the HDBIF Dataset and transform it using many algorithms to make it suitable for BSIF iris recognition.

### 4.1. Dataset Iris Images

The HDBIF Dataset is composed of 1892 iris pictures in .tiff format, coming from 946 different persons. You can observe the various details of the HDBIF dataset in Table 1.

**Figure 2:**
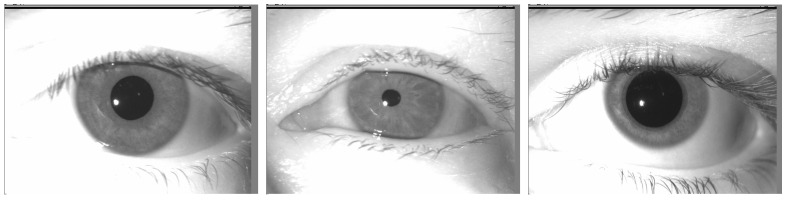
Example of iris images in the HDBIF dataset.

In Figure 2, crucial parameters for iris extraction and unwrapping are observed:

(1) The pupil is centered in the frame: we do not have to center the eye by ourselves.
(2) Part of the iris is under the eyelid and eyelashes. There is also a white spot. Those parts of the iris should not be analyzed in the iris comparison module.
(3) Regarding this database, the radius of the pupil can vary a lot (up to 10×) following the light exposition of the eye. This consideration should be taken into account when searching for circles in the image.

### 4.2. Iris Parameters Extraction (Radius, Center)

Obtaining the main parameters of the eye, including its edges, is primordial to extract and unwrap the iris correctly. For that, we will use the Canny algorithm and the Hough transform.

The Canny algorithm [7], a renowned method in edge detection, identifies precise edges within the image, a critical step for accurate feature extraction. This algorithm not only takes into account the gradient magnitude and direction but also facilitates the extraction of edges with basic functions.

After applying the Canny algorithm, the binary thresholding of the edge image is conducted to isolate significant edge features. The resulting binary image undergoes circle detection executed by the Hough Transform. It is worth noting that there are two variations of the Hough Transform, one designed for lines and another for circles [13]. For the purpose of this study, our focus will be exclusively on circle detection. The Hough Transform, tailored to identify circular shapes in images, is pivotal in detecting the circular boundaries of both the iris and pupil, as well as the iris and sclera. The Hough transform method, as implemented in the Matlab native function imfindcircles, utilizes specific parameters including sensitivity and the radius range of the circles to detect.

The Canny approach effectively identifies potential boundaries of these anatomical features, considering factors like the partial visibility of the iris due to eyelids or eyelashes, and the typically full visibility of the pupil. Consequently, a higher sensitivity setting is used for detecting the iris circle to account for its possible partial appearance in the image. Detected circles are then visualized on the image with different colors, indicating the boundaries between the iris and pupil, and the iris and sclera. The function also calculates the central coordinates of the iris and determines its inner and outer radii based on the detected circular boundaries.

The gradient magnitude and direction are calculated as usual, as detailed below. Let us consider a 2D isotropic Gaussian filter $G_{\sigma }$ of standard deviation σ as in Equation (1):

(1) $$G_{\sigma }\left(x,y\right)=\frac{1}{2\pi \sigma^{2}}\cdot e^{-\frac{x^{2}+y^{2}}{2\sigma^{2}}}\;\;,\;\mathrm{with}\;\sigma \;\in \;R_{+}^{*}\;\mathrm{and}\;\left(x,y\right)\in R^{2}\;\;,$$

(x,y) represent the pixel coordinates in the image. First, the smoothed image $I_{\sigma }$ is obtained by convolution of the original image *I* with the Gaussian $G_{\sigma }$. Then, the image derivatives $G_{x}$ and $G_{y}$ are computed along the *x* and *y* axis in Equation (2).

(2) $$G_{x}=\left[\begin{array}{ccc} -1 & 0 & 1\\ -1 & 0 & 1\\ -1 & 0 & 1 \end{array}\right]*I_{\sigma },\;G_{y}=\left[\begin{array}{ccc} 1 & 1 & 1\\ 0 & 0 & 0\\ -1 & -1 & -1 \end{array}\right]*I_{\sigma },$$

∗ represents the convolution product. The gradient magnitude $|\nabla I_{\sigma }|$ and its direction Θ are computed in Equation (3).

(3) $$\left\{\begin{array}{ccc} |\nabla I_{\sigma }| & = & \sqrt{G_{x}^{2}+G_{y}^{2}},\\ \Theta & = & \arctan\left(\frac{G_{y}}{G_{x}}\right). \end{array}\right.$$

**Figure 3:**
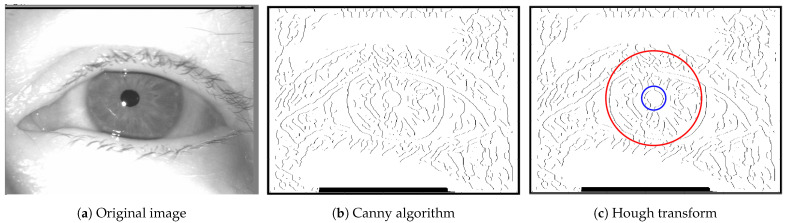
Iris image after Canny algorithm and Hough transform. In (**c**), the blue circle marks the boundary of the interior of the iris, while the red circle marks the exterior.

Finally, the edges are extracted by computing non-maxima suppression in the gradient direction before applying a threshold to obtain binary images, as illustrated in Figure 3b.

In the presented experiments, the Gaussian smoothing in the Canny algorithm takes a standard deviation parameter of σ=7.

The Hough transform detects circles by using the equation of a circle with center (a,b) and a radius of *R*. The equation of a circle is represented in Equation (4).

(4) $${\left(x-a\right)}^{2}+{\left(y-b\right)}^{2}=R^{2}$$

Our approach innovatively extracts the main parameters of the iris using the Iris Parameters Extraction Algorithm detailed in Algorithm 1. This algorithm introduces several advanced image processing steps to ensure high precision and robustness in iris detection. Initially, adaptive Gaussian and median filtering are applied to enhance image quality by reducing noise while preserving crucial details. This is followed by dynamic histogram normalization to adjust image contrast for better edge detection. For the edge extraction process, we employ the Sobel operator which calculates the gradient magnitude and direction at each pixel, ensuring accurate edge detection by highlighting regions with high spatial frequency. This step is critical as it forms the basis for subsequent edge detection and refinement processes. The algorithm then applies directional non-maximum suppression (NMS) to precisely identify true edge points by suppressing non-maximum gradients. Adaptive thresholding is used to employ local image contrast for more accurate edge detection under varying lighting conditions. Finally, morphological closing is performed to reinforce circular outlines, which is crucial for accurate circle detection.

These steps culminate in the Hough Transform applied for precise pupil and iris detection, even under challenging conditions with partial occlusions from eyelashes or varying illumination. As shown in Figure 3c, the inner and outer circles, marked in blue and red, respectively, have centers that are equal to within a few pixels, as defined in the code by a margin of error: $|{\mathrm{center}}_{\mathrm{inner}}-{\mathrm{center}}_{\mathrm{outer}}|\leq \mathrm{error}\_\mathrm{margin}$. This robust detection process distinguishes our method by significantly improving the accuracy and consistency in extracting iris parameters.
**Algorithm 1** Iris parameters extraction**Input:**     HDBIF database image *img***Output:**     Iris inner and outer radius *r_int_*, *r_out_*     Circles center coordinates (*x_int_*, *y_int_*), (*x_out_*, *y_out_*)1:Image smoothing using Gaussian and median filters2:Histogram normalization3:Gradient calculation with Sobel operator4:Apply directional Non-Maximum Suppression (NMS)5:Adaptive thresholding based on local image contrast6:Deleting non-circular components7:Morphological closing     ▹ Reinforce circular outlines8:Hough transform for pupil detection9:**if** ${\mathrm{pupil}}_{\mathrm{center}}-{\mathrm{img}}_{\mathrm{center}}\leq 50$ **then**  ▹ Pupil and image center distance tolerance10:    **if** ${\mathrm{radius}}_{1}\in \left[20;80\right]$ **then**11:        ${\mathrm{pupil}}_{\mathrm{radius}}\leftarrow {\mathrm{radius}}_{1}$12:        ${\mathrm{pupil}}_{\mathrm{center}}\leftarrow {\mathrm{center}}_{1}$13:    **else**14:        Increase sensitivity and repeat detection15:    **end if**16:**end if**17:Crop the image according to pupil size     ▹ Keep iris and avoid error detection18:Hough transform for iris detection19:**if** ${\mathrm{iris}}_{\mathrm{center}}-{\mathrm{pupil}}_{\mathrm{center}}\leq 20$ **then**  ▹ Iris and pupil center distance tolerance20:    **if** ${\mathrm{radius}}_{2}\in \left[100;180\right]$ **then**21:        ${\mathrm{iris}}_{\mathrm{radius}}\leftarrow {\mathrm{radius}}_{2}$22:        ${\mathrm{iris}}_{\mathrm{center}}\leftarrow {\mathrm{center}}_{2}$23:    **else**24:        Increase sensitivity and repeat detection25:    **end if**26:**end if**27:**if** max sensitivity reached **and** no iris found **then**28:    Define ${\mathrm{iris}}_{\mathrm{radius}}$ based on iris size estimation29:**end if**30:Round ${\mathrm{iris}}_{\mathrm{radius}}$ and ${\mathrm{iris}}_{\mathrm{center}}$ coordinates     ▹ For future use

The innovative combination of these techniques ensures that the detected parameters of the iris are precise and reliable, setting a solid foundation for subsequent biometric analysis.

### 4.3. Iris Extraction

Now that the main parameters of the iris were extracted (radius and center coordinates), we have to extract the iris from the image. The procedure begins with the establishment of a storage folder for processed images. The extraction of essential parameters, including the iris’s external and internal radii and the eye’s center coordinates, are explained in Section 4.2.

**Figure 4:**
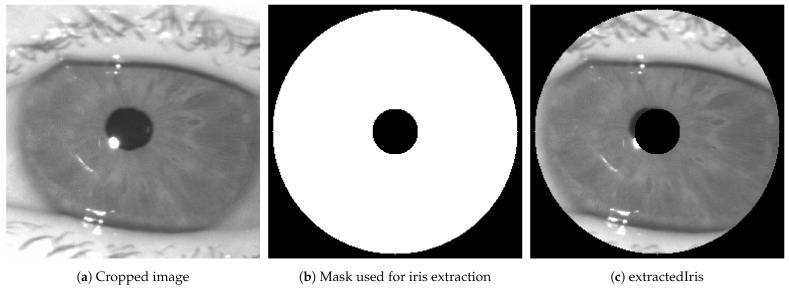
Extracted iris using Canny algorithm and Hough transform to create the filter.

Following the display of the original image, a cropping operation focuses on the eye region, ensuring the entire iris is included. Then, a filter that isolates the iris ring is created. This filter, designed based on the calculated radii and center, effectively forms a circular mask that targets the iris. The iris is extracted by multiplying the cropped image with this filter, resulting in an isolated and clear representation of the iris. This process, detailed in Algorithm 2 and pictured in Figure 4, not only accentuates the iris for detailed analysis but also prepares it for applications such as biometric identification using BSIF detailed in Section 5, where the iris’s unique patterns are crucial for individual identification.
**Algorithm 2** Iris extraction**Input:**     Image from the HDBIF database *I*     Iris characteristics (inner and outer radius, center of the circles)     Coordinates of the center of the circles $\left(x_{center},y_{center}\right)$**Output:**     File path to the extracted iris image     Cropped squared image around the eye     Filter used to extract the iris     Extracted iris1:File path creation2:Crop image     ▹ Faster calculations3:Copped image coordinates: ($x_{\min\;\max}$,$y_{\min\;\max}$) = center (${\mathrm{outer}}_{\mathrm{radius}}$ + margin)4:Filter extraction5:$\mathrm{filter}\left(i,j\right)=\left(r_{\mathrm{inner}}\leq \sqrt{{\left(i-x_{\mathrm{center}}\right)}^{2}+{\left(j-y_{\mathrm{center}}\right)}^{2}}\leq r_{\mathrm{outer}}\right)$6:${\mathrm{extracted}}_{\mathrm{iris}}\leftarrow I\cdot \mathrm{filter}$

### 4.4. Iris Unwrapping

The final script in the preprocessing stage is the unwrapping process. This script transforms the circular iris region into a rectangular form, permitting the application of Binary Statistical Image Features (BSIF) [8] in the recognition stage. The unwrapping process ensures that the features of the iris are presented in a consistent and analyzable format. In the second part of the process, we will apply a double threshold to the image to create a binarized mask that will define the pixels analyzed by the matching code part of the second module.

**Figure 5:**
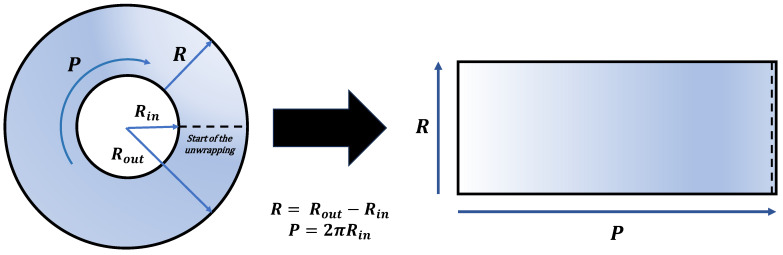
Unwrapping process: from Polar (**left**) to Rectangular coordinate (**right**).

Initially, the length of a rectangle, intended to encapsulate the unwrapped iris, is determined based on the iris’s radius. Coordinate arrays are created to define the spatial points of this rectangle. Central coordinates of the eye are specified, and an angle vector is initialized to assist in generating perimetric points, both inner and outer, around the eye’s circumference. These points are then used to create the lines of the rectangle using linear interpolation, showcased in Figure 5, effectively unwrapping the iris into a flat, rectangular image. This image is displayed and resized to a new dimension for compatibility with a specific filter.

Furthermore, a mask is constructed by setting thresholds on the pixel values of the iris image, creating a binary representation that highlights certain features of the iris. This mask is visualized, converted to a logical array for filter compatibility, and then stored as an image file. The combination of geometrical calculations, image preprocessing techniques, and visualization tools allows for a comprehensive approach to iris feature extraction and representation.

Each of these scripts, detailed in Algorithm 3, contributes significantly to the preprocessing stage, ensuring that the iris images are optimally prepared for accurate and efficient recognition.
**Algorithm 3** Iris unwrapping**Input:**     Extracted iris     Inner and outer radius of the iris (innerRadius,outerRadius)     Coordinates of the center of the circles $\left(x_{center},y_{center}\right)$**Output:**     Unwrapped iris img     Mask of the unwrapped iris mask1:Unwrapped iris rectangle: $size\left(\mathrm{rect}\right)=\left(2\pi \cdot {\mathrm{inner}}_{\mathrm{radius}}\right)\times \left({\mathrm{outer}}_{\mathrm{radius}}-{\mathrm{inner}}_{\mathrm{radius}}\right)$2:Generation of perimetric points:3:**for** i=1 to length(rect) **do**4:    $\left(x_{\left(1,2\right)}\left[i\right],y_{\left(1,2\right)}\left[i\right]\right)={\mathrm{center}}_{1,2}+\mathrm{inner}\mathrm{Radius}/\mathrm{outer}\mathrm{Radius}*\cos\left(\theta \left[i\right]\right)$5:**end for**6:Generation of rectangle lines using interpolation:7:numPoints = outerRadius−innerRadius8:**for** i=1 to length of the rectangle **do**9:    ($x_{line}$, $y_{line}$) ← linspace($\left(x_{1},y_{1}\right)\left[i\right]$, $\left(x_{2},y_{2}\right)\left[i\right]$, numPoints)10:    $y_{line}$← linspace(y1[i], y2[i], numPoints)11:    Linearly interpolate pixel values in positions given by $x_{line}$ and $y_{line}$12:    Assign interpolated pixel values to $i^{th}$ column in unwrapped iris image img13:**end for**14:Double thresholding to obtain iris mask:15:Calculate mean pixel value of the unwrapped iris image.16:($lower_{thresh}$, $higher_{thresh}$) ← (0.5×mean, 1.3×mean)17:Find pixels in unwrapped iris image where $pixel_{value}$ is within threshold range:18:**if** $lower_{thresh}$ < $pixel_{value}$ < $higher_{thresh}$ **then**19:     $mask_{pixel}$← 1.20:**end if**

### 4.5. Examples of Iris Images: Before and after Preprocessing

**Figure 6:**
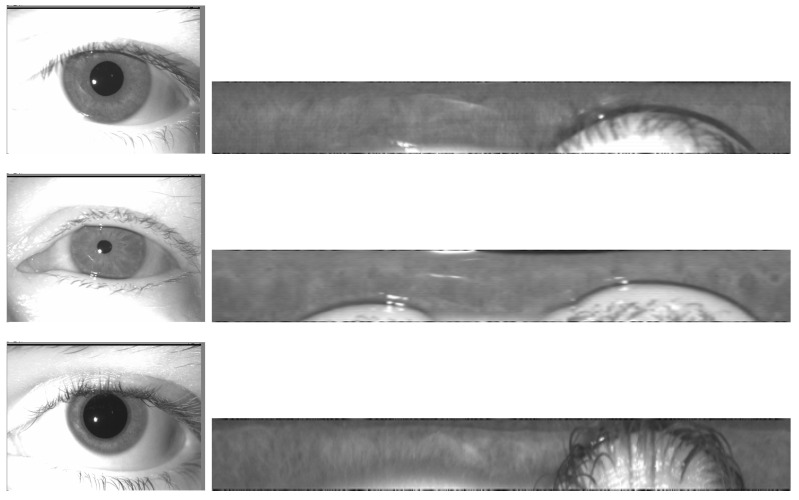
Iris images (**left**, size 640 × 480) and their unwrapping (**right**, size 512 × 64).

To demonstrate the efficacy of our preprocessing techniques, we present examples of iris images before and after the application of our pre-processing scripts. These examples displayed in the Figure 6 illustrate the significant improvements in image quality and clarity, crucial for accurate iris recognition.

## 5. Iris Recognition

The iris recognition module heavily relies on the groundbreaking work of researchers from the University of Notre Dame in the field of iris biometrics [12]. This work plays a crucial role in the development of our module’s scripts and algorithms.

A key point of our methodology is the application of the Binarized Statistical Image Feature (BSIF) [8] for identity verification through the analysis of pre-processed iris images. The BSIF method employs statistical machine learning to identify key textural patterns in the iris. It computes binary codes for each pixel by projecting local image patches using linear filters and then binarizing these projections. The length of these binary codes is determined by the number of filters used, and the resulting histograms of these codes are instrumental in recognizing textural patterns, thus creating a unique binary representation of these features.

The BSIF filtering starts by obtaining the filter response with Equation (5):

(5) $$s_{i}=w_{i}^{⊤}x$$

$s_{i}$ represents the response of a particular filter. The filter itself is denoted by $w_{i}$, and x is the local image patch under analysis. This equation computes the response of the image patch to a specific filter.

After this, the process continues by combining the different filter responses:

(6) s=Wx

In Equation (6), *s* is the collective response vector obtained from applying all filters. W represents the matrix of all filters used, and x continues to represent the image patch in the various equations. This equation combines the responses from multiple filters to create a comprehensive response profile for the image patch.

Finally, the following matrix undergoes preprocessing by combining the different filter responses in Equation (7):

(7) $$V=D^{-1/2}E^{⊤}\;$$

Here, D and E are obtained from the eigendecomposition of the covariance matrix and are integral in constructing V.

This innovative approach, which involved the creation of domain-specific BSIF filters trained on iris-specific data rather than generic natural scenes, has proven to be more effective than traditional methods. The findings indicate that these domain-specific BSIF features outperform standard ones, showcasing the effectiveness of this tailored approach. Furthermore, they demonstrated the importance of how training data are selected, showing that image patches chosen based on human performance in eye-tracking experiments yield better results than randomly selected patches [12]. In this process, humans were presented with pairs of iris images and asked to determine whether the irises belonged to the same person or different individuals. By analyzing their eye movements, researchers identified the specific patches of iris images that humans focused on to make their decisions. These patches were then selected and incorporated into the BSIF algorithm. This method of data selection, guided by human-task performance, proved to be significantly more effective than randomly selected patches by a computer, yielding better results in iris recognition. This novel approach is a first in the field and contributes significantly to the advancement of iris recognition technology.

### 5.1. Extraction of the Binary Code

In the binary code extraction process for iris recognition, a series of steps detailed in Algorithm 4 are followed to transform iris images into a binary format that is essential for identification. Initially, iris images are loaded, including specific masks corresponding to each iris, focusing on fundamental features while filtering out unnecessary parts. The images are then “wrapped” to ensure the uniform application of texture filters across all images, a critical step for consistent feature extraction. Next, specific filters tailored for iris texture are selected. These filters are then applied to the iris images, capturing the unique textural patterns of each iris. The output from this filtration is then converted into binary codes, representing the distinctive features of the iris. This binary representation is crucial as it forms the foundation for the subsequent matching and recognition processes in iris biometrics. The code effectively distills the complex patterns of the iris into a format that can be efficiently compared and analyzed, playing a key role in the accurate identification of individuals based on their unique iris patterns.
**Algorithm 4** Iris binary code extraction**Input:**     Unwrapped iris img (see Algorithm 3)     Texture filters**Output:**     Unwrapped iris binary code1:Load unwrapped images, masks and iris texture filters2:Number of kernels = numScl     ▹ size(texturefilters,3)3:Wrapping of the image:     r←size(texturefilters,1)/2    ▹ Size of the filters     $up_{img}=img\left(1:r,:\right);\;bt_{img}=img\left(\left(end-r+1\right):end,:\right);$    ▹ Sides     $lf_{img}=img\left(:,1:r\right);\;rt_{img}=img\left(:,\left(end-r+1\right):end\right);$     $cr_{11}=img\left(1:r,1:r\right);\;cr_{12}=img\left(1:r,\left(end-r+1\right):end\right);$    ▹ Corners     $cr_{21}=img\left(\left(end-r+1\right):end,1:r\right);\;cr_{22}=img\left(\left(end-r+1\right):end,\left(end-r+1\right):end\right);$
4:Loop over the kernels:5:**for** i=1 to numScl **do**6:    Select and apply the $numScl_{th}filter$7:    $codeBinary=numScl_{th}filter\left(wrappedImg\right)>0$    ▹ Generate binary code by thresholding the $i_{th}$ numScl filter applied to the wrapped img8:**end for**

### 5.2. Matching of the Binary Code

**Figure 7:**
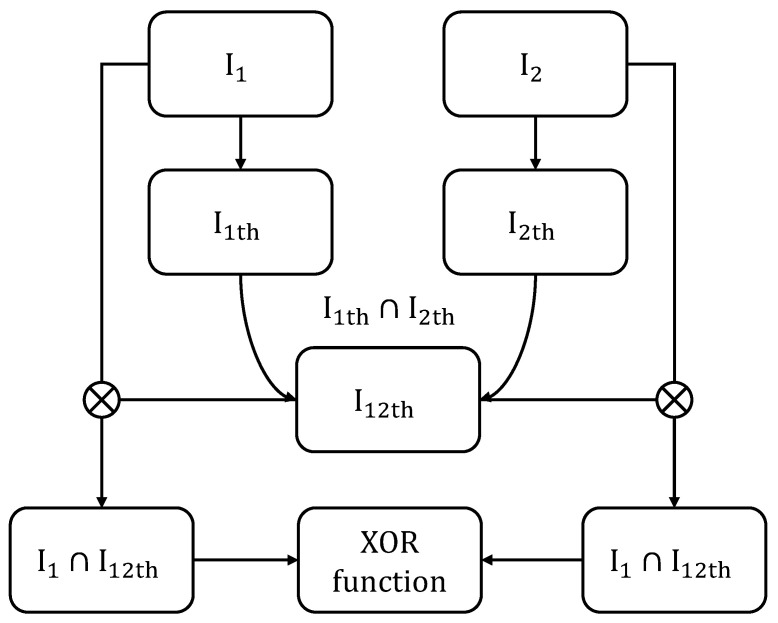
Score comparison process. $I_{1\mathrm{th}}$ and $I_{12\mathrm{th}}$ represent binarized iris images due to thresholding. The intersection (∩) represents element-wise multiplication.

The process showcased in Figure 7 and detailed in Algorithm 5 starts by adjusting the binary codes to ensure an effective comparison. This involves removing the margins that were added for the extraction of the binary code.
**Algorithm 5** Iris code matching**Input:**     Binary codes of both irises     Masks of both irises**Output:**     Iris similarity comparison score1:Remove margins2:Multiply both iris masks: $mask=mask_{1}.*mask_{2}$3:Initialize a score matrix $Score_{\mathrm{bit},\;\mathrm{shift}}$4:Initialize the range of possible shifts $Max_{shift}$5:**for** shift from $-\max_{shift}$ to $\max_{shift}$ **do**6:    **for** each bit in the binary code **do**7:        $\mathrm{XOR}\left(codeBinary1_{bit}\right),\mathrm{shift}\left(codeBinary2_{bit}\right)$8:        Apply masks and use bitwise AND to compare essential parts of the iris codes9:        Filtering of resulting XOR values using an AND mask10:        ${Score}_{\mathrm{bit},\;\mathrm{shift}}=\frac{\sum (\mathrm{XOR}\left(codeBinary1_{\mathrm{bit}},\mathrm{shift}\left(codeBinary2_{\mathrm{bit}}\right)\right)\;\mathrm{AND}\;\mathrm{mask})}{\sum (\mathrm{mask})}$    ▹ The matrix contains comparison scores for each bit and for each shift11:    **end for**12:**end for**13:Score = min(mean(Score))    ▹ Min avg score for optimal rotation matching across kernels

The system then multiplies both masks of the images to analyze the similarities between irises (as the role of the mask on each picture is to mask regions of the extracted cylinder which are not iris, such as eyelashes or eyelids, as illustrated in Figure 6). After this, for each section of the binary code, the system performs a comparison with a corresponding section from another iris’s binary code. This comparison is designed to identify similarities between the two codes. The similarity is quantified based on how many elements of the codes match by computing a score ${\mathrm{Score}}_{\mathrm{bit},\;\mathrm{shift}}$:

(8) $${\mathrm{Score}}_{\mathrm{bit},\;\mathrm{shift}}=\frac{\sum (\mathrm{XOR}\left(codeBinary1_{\mathrm{bit}},\mathrm{shift}\left(codeBinary2_{\mathrm{bit}}\right)\right)\;\mathrm{AND}\;\mathrm{mask})}{\sum (\mathrm{mask})}.$$

One of the unique challenges in iris recognition is the natural rotation of the eye between different images. To address this, the system includes a mechanism to adjust for eye rotation. This is conducted by shifting one of the binary codes slightly in different directions and comparing these shifted versions to find the best alignment as showcased in Equation (8).

The result of this comparison is a score reflecting the degree of similarity between the two iris images. A lower score (Equation (8)) indicates a higher likelihood that the two images are from the same iris, while a higher score suggests they are from different individuals. This score is crucial in applications like security systems, where it is essential to accurately determine an individual’s identity.

To further refine the results, the system calculates comparison scores for different scenarios, such as comparing two images from the same eye (genuine comparison) and comparing images from different eyes (impostor comparison). These scores provide valuable insights into the effectiveness of the iris recognition system and help in fine-tuning its accuracy and reliability.

### 5.3. Illustrating the Iris Code Comparison Process

A critical aspect of iris recognition is the comparison of iris codes extracted from different images. This process is essential for determining an individual’s identity based on their unique iris patterns.

The iris codes, extracted through specific algorithms, are binary representations of the iris textural patterns. The comparison of these binary codes evaluates the degree of similarity by calculating a similarity score based on the number of matching bits between the codes, as shown in Equation (8). A higher similarity score indicates a closer match, suggesting that the two iris images likely belong to the same individual.

One of the challenges in iris recognition is the rotational variance of the iris in different images. To address this, the comparison algorithms include mechanisms to compensate for eye rotation by allowing a degree of shift in the binary code comparison. These techniques together ensure that the iris recognition process is not only accurate but also resilient to common variations in iris images, such as rotation and positional shifts.

## 6. Results

The efficiency of the proposed methodology was quantified through statistical analysis conducted on two files: the first containing pairs of iris images known to belong to the same person, and the second containing pairs of iris images known to belong to different people. The aim was to determine if these results were preserved after applying our iris extraction method.

**Figure 8:**
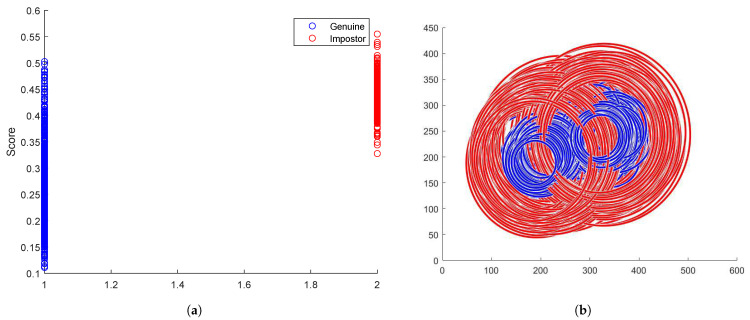
Score distributions and radii/position distributions for iris recognition. (**a**) Genuine (blue) and Impostor (red) scores distribution. (**b**) Pupil (blue) and Iris (red) radii and position distribution.

The database was initially subjected to a comprehensive unwrapping procedure. Following this, a meticulous line-by-line analysis was conducted on both files. The BSIF iris comparison algorithm was then applied to the specified pairs of irises mentioned in each respective line. This facilitated the acquisition of comparison scores for each iris pair enumerated within the files. Consequently, distributions of scores for genuine and impostor iris pairs were established, enabling a rigorous evaluation of the identification accuracy, as illustrated in Figure 8a. You can also see in Figure 8b the distribution of pupil and iris radii sizes and positions.

The mean, standard deviation, and variance for each distribution were subsequently computed, culminating in the presentation of the data within Table 2.

Employing the various metrics delineated in the preceding table, in conjunction with the visual representations of score distributions, a threshold demarcating the distinction between pairs of iris images corresponding to the same individual versus those that do not, was established at 0.385. This threshold enables the calculation of the error percentage attributable to the preprocessing of iris images, which is reported in Table 3.

These results averaging 4.93% global errors validate the robustness and reliability of our proposed method in accurately preprocessing and recognizing iris images, demonstrating its potential for real-world biometric security applications.

## 7. Discussion

This article has presented a comprehensive study on the preprocessing and recognition of iris images, highlighting significant contributions to the field of biometric security and offering insights into future research directions. The innovative methodologies applied to the preprocessing of iris images have significantly enhanced the quality and uniformity of the data, setting a solid foundation for accurate biometric analysis. Through meticulous cleaning, edge detection, and transformation techniques, we have successfully addressed the inherent complexities associated with iris recognition, such as occlusions and variations in pigmentation and texture.

The proficiency of the Canny edge detector algorithm combined with the circle Hough transform in delineating the intricate structures of the iris has been demonstrated, proving their efficacy in extracting the vital characteristics required for reliable identification. The unwrapping and masking strategies employed further refine the process, ensuring that the subsequent BSIF analysis operates on data of the highest integrity.

In conclusion, the advancements made in this study, particularly in the preprocessing phase, represent a significant step forward in the field of iris recognition. However, the journey does not conclude here. The continuous evolution of image processing techniques and the integration of machine learning will undoubtedly open new avenues for research, promising even greater accuracy and efficiency in biometric identification systems.

### Limits and Potential Improvements

As seen in Table 3, the iris extraction and unwrapping process is very reliable for BSIF iris comparison. Despite the advancements presented in this work, there remain areas where the preprocessing methodology may be further enhanced. While the preprocessing algorithms have demonstrated efficacy, they are not without constraints. One such limitation is the assumption of ideal conditions within the iris images. Real-world scenarios may introduce variables, such as occlusions or varying lighting conditions, that could impede the accuracy of the feature extraction process. Additionally, the present algorithms may not adequately account for the high variability in iris pigmentation and texture across different populations. Future iterations of this methodology could benefit from machine learning techniques paired with a broader dataset that includes a wide range of iris conditions and variations. In summary, while the presented method constitutes a substantive stride in iris recognition technology, continuous efforts to address the outlined limitations and incorporate the suggested improvements could yield a more versatile and error-resilient system.

## Figures and Tables

**Table 1:** HDBIF Iris dataset parameters.

Parameters	Value
Number of images	1892
Number of same iris pairs	946
Number of different iris pairs	473
Format	.tiff
Size	640 × 480
Number of channels	3
Quantification	3 × 8 = 24 bits

**Table 2:** Iris distributions metrics.

Distribution	Genuine	Impostor
Mean	0.26482	0.44308
Standard Deviation	0.069778	0.03163

**Table 3:** Iris distributions error percentages.

Distribution	Genuine	Impostor
Number of errors	54	16
Number of pairs	946	473
Error %	5.7143%	3.3898%

## Data Availability

The HDBIF dataset presented in this research article is available on the University of Notre-Dame Datasets (https://cvrl.nd.edu/projects/data, accessed on 16 July 2024). The code used for the different transformations can be found in the associated GitHub repository (https://github.com/arthur-ru/Iris-Images-Preprocessing-for-BSIF-Iris-Recognition, accessed on 16 July 2024). Any additional data, if required, can be obtained by contacting the corresponding author upon request.

## Abbreviations

The following abbreviations are used in this manuscript:

BSIF	Binarized Statistical Image Features
HDBIF	Human-inspired Domain-specific Binarized Image Features
